# An eye- tracking technology and MLP-based color matching design method

**DOI:** 10.1038/s41598-023-28331-7

**Published:** 2023-01-23

**Authors:** Yinhong Hua, Jianlin Ni, Honglei Lu

**Affiliations:** 1 School of Fine Arts, Nanjing Normal University, Nanjing, 210000 China; 2 College of Arts, Anhui University, Hefei, 230039 China

**Keywords:** Energy infrastructure, Mechanical engineering

## Abstract

Images are a significant source of inspiration for designers to carry out the color design. However, the absence of animated images in the product color design can create confusion for designers. To translate the colours of the animated images into product colours, this work used eye-tracking technology to aid colour extraction and the multilayer perceptron neural network (MLP) algorithm to train a product colour decision model to filter the best product colour schemes. Firstly, eye tracking technology is used to collect the distribution of hotspots of the subject while viewing the animated images. Based on the distribution of eye-tracking hotspots, the most interesting animated colours were extracted. Then, the MLP is applied to train a colour decision model for children's shopping cart products, and the colour decision model is used to filter the optimal solution for the product colour, and finally the colour design is completed from the animated colour to the three-colour children's shopping cart product. Experimental results show that the color extraction based on the eye-tracking technology and the color scheme screening based on the intelligent algorithm can realize the effective conversion from animated image colors to product colors. This work proposes a color scheme design method from animations to products, which further expands the image color sources in product color design and can accurately find the color scheme that matches the animated image and the product.

## Introduction

Color, as a major factor affecting vision, plays a key role in determining consumers' satisfaction with product appearance^1^. A reasonable product color scheme can capture consumers' visual attention, enhance their psychological identity and sense of pleasure to the product, which further influence consumers' brand judgment and purchase decisions on products^2,3^. To this end, many scholars have carried out research in different aspects, including image color reuse^4^, product color planning^5^, product color evaluation^6^, product color emotion^7^, product color imagery^8^, product color aesthetics^9^, and color trend prediction^10^. Among them, the image color reuse is to extract the flat image color style in the process of product design, where the flat images is an important source of inspiration for designers to carry out product color scheme design^4^. However, consumers will expect more different color combinations based on product categories or color trends, which can be a difficult job for designers^11^. Animation is a formal of visual communication^12^. In addition, the animated image colors are more likely to remind consumers of favorite images in their hearts, thus improving the satisfaction with color matching^13^. Therefore, carrying out research on converting animated image colors to product colors can quickly and accurately find the color schemes that match animated images and products, which is of great significance for further expanding the source of image colors in product color design^4^.

Currently, the research on image color reuse can be roughly divided into the following two aspects^3,14^. One is to map the target product color scheme by referring to the color styles of plane images^3^. Researchers usually draw on the colors of various plane images and extract colors through random extraction, cluster analysis and other approaches, and then provide references for designers in the process of product color design^15–20^. The other is to realize the mapping to the target product color scheme according to the image color semantics of plane images^14^. As is known to all, it is an important task to mine consumers' perception of colors, establish mapping relationship between consumers' emotions and colors, and further convert emotional images to product color design elements^14,21,22^.

In summary, the extraction of image colors has become a hot topic in color research. Numerous scholars have carried out several studies on the extraction and reuse of image colors, and even the situations where the brand color of an international company or organization is restricted have also been considered^23,24^. These studies provide a wide range of color sources for product color design and plays important roles in satisfying consumers' color preferences and promoting consumers' purchasing desires.

Nevertheless, there remain two overlooked problems to be solved. Firstly, consumers will expect more colors with different imagery^11^. Since source images from different dimensions can provide more inspiration for the target color scheme^13^, the lack of consideration of existing color cases with more dimensions will inevitably limit the success rate and efficiency of product color matching to a certain extent^3^. As a form of expression of visual communication, Animation leaves a deep impression on consumers. Extracting colors from animated images can more easily remind consumers of favorite images in their hearts, thereby improving consumers' satisfaction with color matching^13^. Secondly, the choice of numerous product color schemes is a difficult decision for designers. For example, even colors extracted from a single image can have hundreds of color combinations. At this time, it is necessary to establish an decision model between consumers' emotional preferences and product colors^17,25,26^.

Therefore, this work proposes a color scheme design method from animations to products, which further expands the image color sources in product color design and can quickly and accurately find the color scheme that matches the animated image and the product. The proposed method also contributes to helping designers to quickly complete the product color matching design work. This work uses eye-tracking technology to extract and reuse the colors of animated images in product color schemes, which is an important innovation in the field of color research and application.

## Results

### Colour extraction of animated images based on eye tracking technology

**Figure 1:**
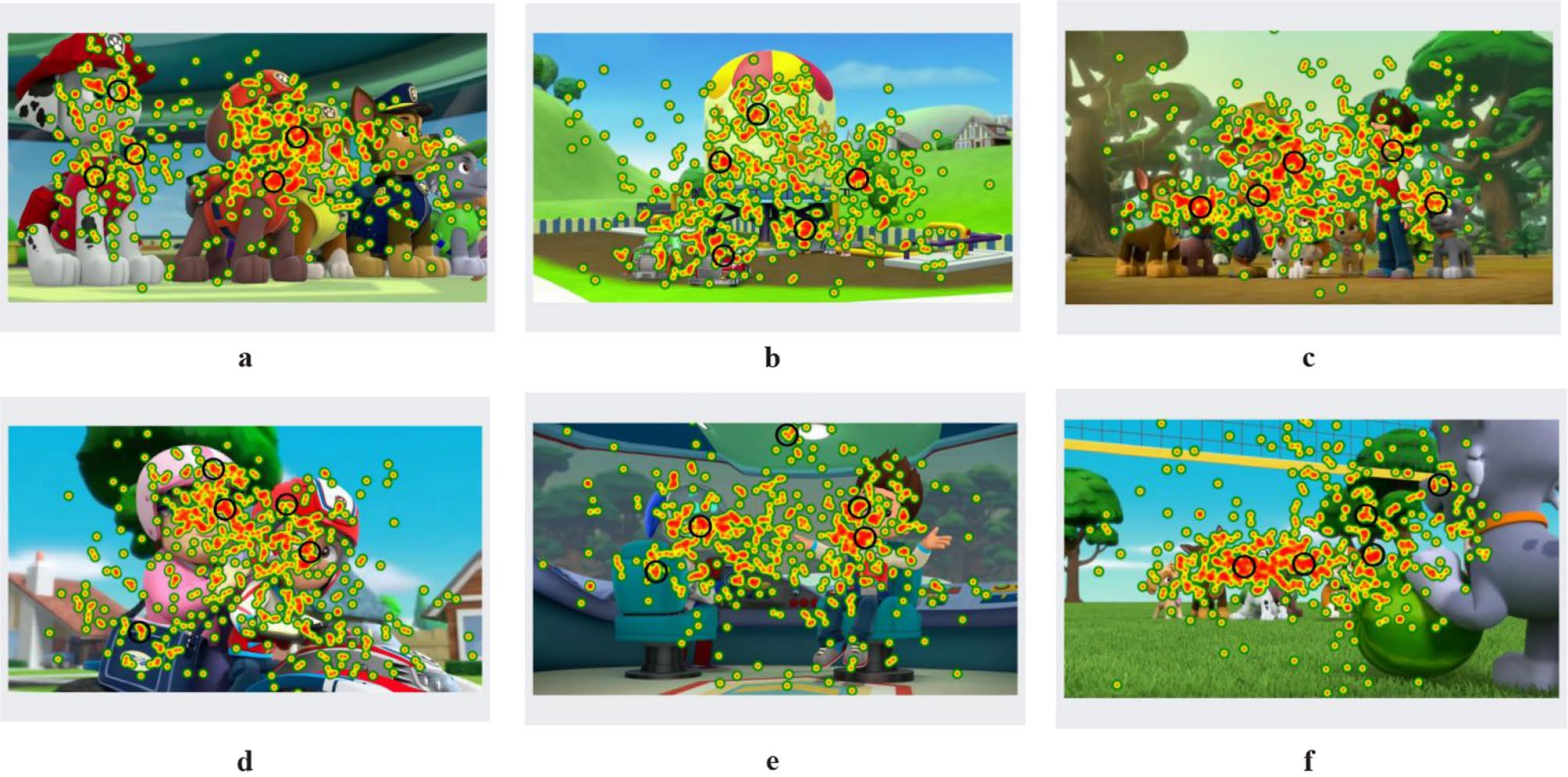
Animated image eye-tracking hot zone map.

Animated image color extraction is performed using Tobii Pro Glasses 2 eye-tracking device for data acquisition, and the thermal area map is shown in Fig. 1. Figure 1 was generated using ErogLAB 1.0 software on Tobii Pro Glasses 2 eye-tracking device (ErogLAB 1.0, http://www.ergolab.cn/products/modules/eyetracking) and Adobe Photoshop 2022 software (Adobe Photoshop 2022, https://www.adobe.com/cn). /products/photoshop.html). Tobii Pro Glasses 2 eye-tracking device is made in Tobii with a sampling frequency of 50 Hz and a field of view of 160° (horizontal). It also supports automatic parallax correction and slip compensation. The experiment is conducted in the Interactive Science Laboratory of Anhui University with 20 children, 11 boys and 9 girls (Average age: 7 years old). 20 subjects enter the laboratory in turn and put on the eye-tracking device while keeping a fixed distance from the monitor. After calibrating the position of the subject's eye observation, the experiment start and data are collected at the same time. If the eye-tracking device can not accurately locate the participant's eyes, the participant will not be allowed to participate this experiment. All subjects have signed an informed consent form before the experiment and receive some material rewards when the experiment completes.

As shown in Fig. 1, the hot areas captured by eye-tracking device are mainly concentrated in the subject where the color of characters is concentrated on the face, clothing and seat. This indicates that it is indeed easy for people to look at the animated images they like in hearts, which is consistent with the findings of Xu.^13^ However, different from Hsiao et al.^19^ and Ding et al.^21^, the eye-tracking technology extracts a more intuitive heat zone map. Besides, the adoption of eye-tracking heat zone maps avoids the calculation of complex formulas such as color clustering, which reduces the requirement for expertise in color reuse of animated images and improves the efficiency of product color design^11,16,19,21^.

**Figure 2:**
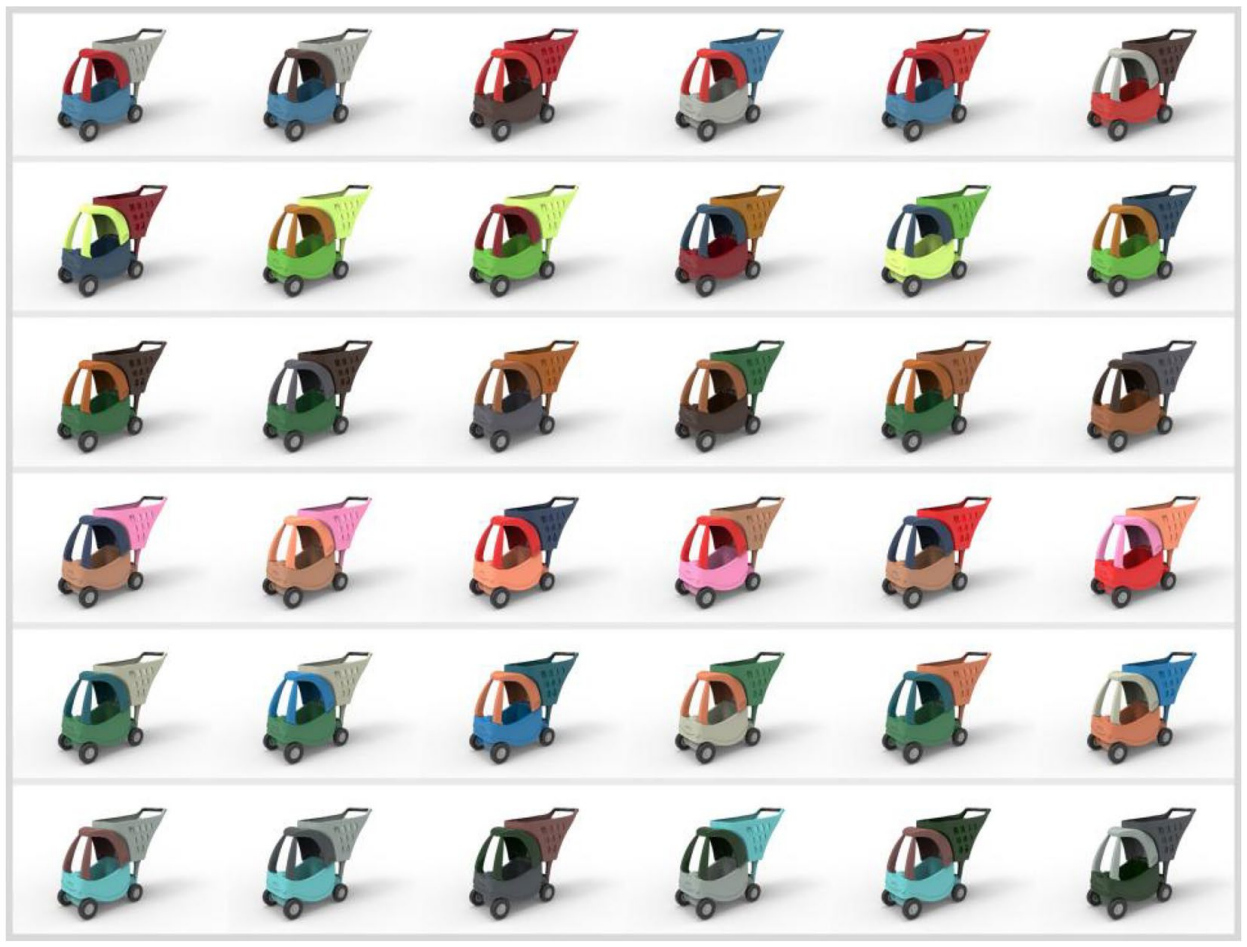
Part of the 360 color design schemes of the three-color children's shopping cart.

Finally, a total of 30 colors are extracted from 6 animated images. Each image is taken as a group and 5 colors are extracted for each group, specific color groups and color HSB parameter values are shown in Table 1. The hot spots in the eye-tracking heat zone map are sorted according to the size of their relative distribution. The top 5 colors (from largest to smallest order) are absorbed using PS software and their HSB values are recorded. Specific color extraction area is shown in the black circle of Fig. 1. Since most of the animated image colors give up the transition between colors, a minor change in the color extraction position will not have a large impact on the colors^12^. Each group has 5 different colors and the samples that need to be color-designed only contains 3 colors, so each group can design 60 different product color schemes by random combination. Eventually, 6 groups of 360 color combination schemes for the children's shopping chart are generated through Keyshot 6.0 software (Keyshot 6.0, https://www.keyshot.com/). Some color design schemes are shown in Fig. 2.

**Table 1:**
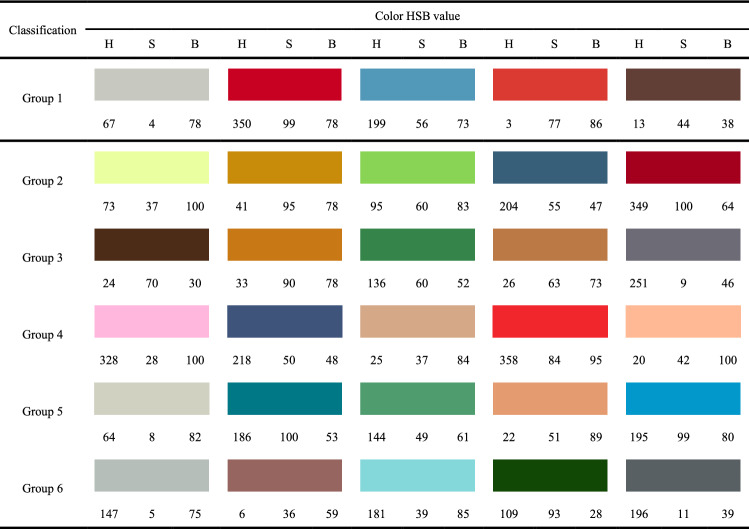
Product color grouping and color HSB parameters.

In Fig. 2, renderings of part of the color scheme of the three-color children's shopping cart are presented, which suggest that the eye-tracking technology can be applied to extract colors from animated images for product color design. Only 6 animated images are selected for color extraction demonstration in this paper. If more animated images are used, the number of output schemes will be far more than 360. Therefore, it is a time-consuming task for designers to select an excellent scheme from the huge number of color schemes, which is also proved by Li et al.^17^ According to Liu et al.^16^, Hsiao et al.^19^, Ding et al.^1^ and Yang et al.^22^ an evaluation mechanism between consumers' emotional preferences and product colors is needed for to color scheme evaluation and decision-making.

### Product colour decision model training and colour selection based on MLP algorithm

Three-color children's shopping cart color decision is a color scheme selection based on MIP. The decision process contains three main steps, data research, model training and result output.

During the data research phase, 360 different product colour schemes were numbered 1–360 and a subjective questionnaire was developed for each of the 360 product colour schemes. 100 children aged 4–9 and their parents in this study were asked to rate the 360 product colour schemes according to their subjective preference. Scores were given on a percentage scale, with 30–60 representing a poor product colour scheme, 60–85 representing a medium product colour scheme and 85–100 representing a very good product colour scheme. The 100 valid questionnaires were collected, and then the variance analysis function of SPSS software was used to eliminate some questionnaires with obvious errors. Then the schemes with an average score of more than 85 were rated as "excellent", and those with an average score of less than 85 were rated as "poor". Finally, a rating result table containing 360 product color schemes was formed. The table of 360 product colour schemes can be accessed from the website (Data in supplementary information). Of these 360 items, the first 300 were used to train the product colour decision model, while the remaining 60 were used to validate the product colour decision model.

In the model training phase, the multilayer perceptron neural network module of IBM SPSS Statistics 26.0 software was used to train and construct the colour decision model for the children's shopping cart products. SPSS software, together with Matlab software, GNU Octave software and Scilab software, can be applied to the training and evaluation of colour decision models. Although the inherent parameter setting of SPSS software limits the logical thinking of users, and the analysis process needs to occupy a lot of computer resources, SPSS software has the advantages of comprehensive function, easy to learn and use, and powerful data access ability. Compared with other software, SPSS does not need to write grammar code, which is suitable for designers to use when carrying out complex mathematical calculations. Subjectivity plays an important role in color decision making, especially the advice of experts. Therefore, the model trained in this study will give multiple optimal product color design schemes, and designers or design experts can quickly obtain the final optimal product color scheme as long as they manually select the schemes given by the model^27,28^^.^ The HSB data extracted from Table 1 and the evaluation results obtained from the questionnaire are taken as the independent variables and the dependent variables, respectively. The Cook distances of the data are firstly analyzed and scores that are obviously wrong are eliminated, and then the MLP is trained. The training process will stop when the loss function doesn't decrease in two consecutive steps.

**Figure 3:**
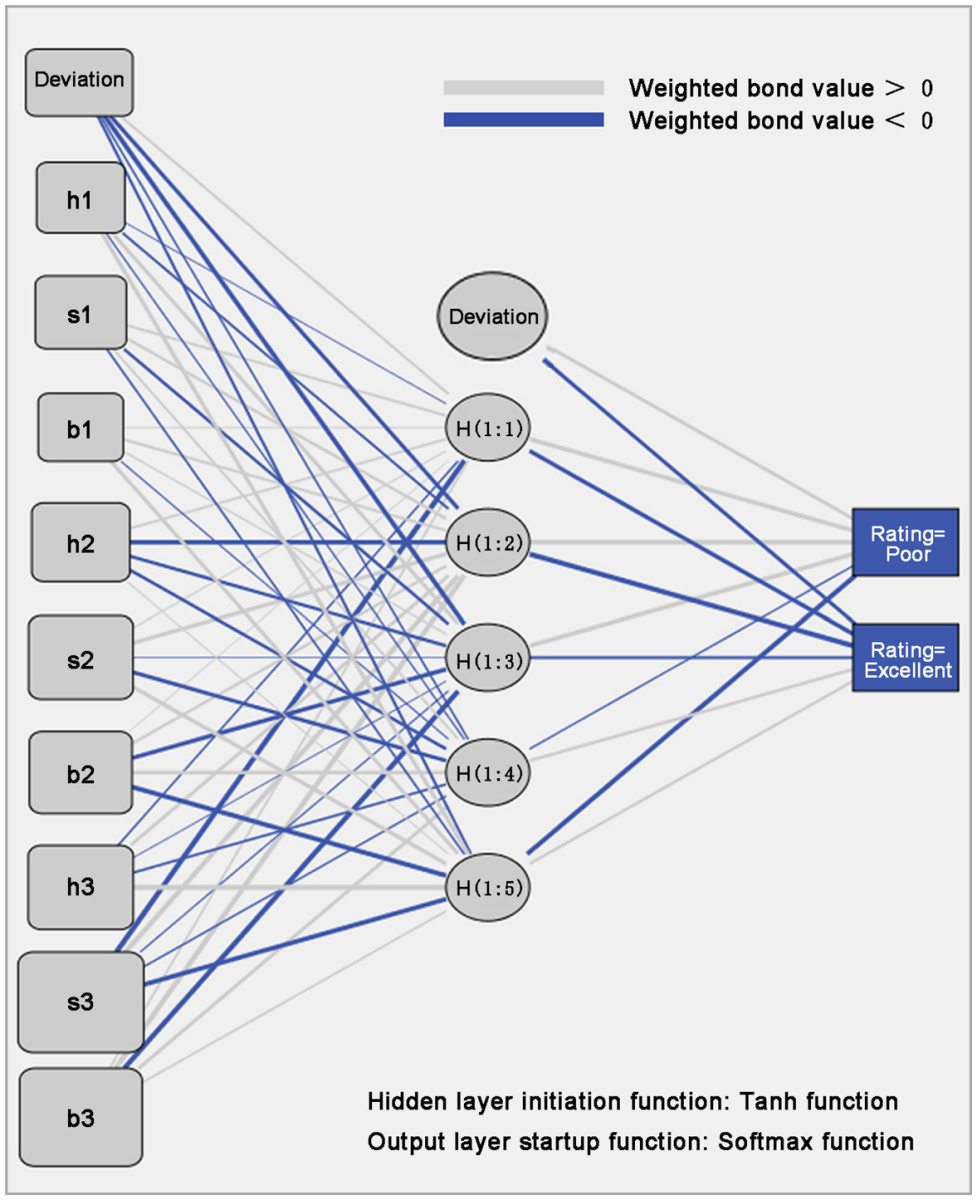
Network structure of the completed training child shopping cart product color decision model.

After repeated training and adjustment, the basic parameters of the trained children's shopping cart product color decision model are shown in Table 2. The structure of the trained children's shopping cart product color decision model is shown in Fig. 3 (Fig. 3 is generated by IBM SPSS Statistics 26 software. IBM SPSS Statistics 26, https://www.ibm.com/products/spss-statistics), where the color of the connection line represents the positive and negative weights, and the thickness of the connection line represents the size of the weights. The input layer to hidden layer connection weights and bias values of the trained children's shopping cart product color decision model are shown in Table 3. The weights and bias values of the connection from the hidden layer to the output layer of the children's shopping cart product color decision model are shown in Table 4. Based on Tables 3, 4, 5 and Fig. 3, a product color decision model is constructed as shown in Eq. (1).

**Table 2:** Basic parameters of MLP model.

Parameters	Value
Initial learning rate	0.4
Number of input data	9
Number of hidden layers	1
Number of neurons in the hidden layer	5
Number of neurons in the output layer	2
Hidden layer activation function	tanh function
Output layer activation function	softmax function
Optimization algorithm	Gradient descent method

**Table 3:** Input layer to hidden layer connection weights and bias values.

	*h*_1_	*h*_2_	*h*_3_	*h*_4_	*h*_5_
Offset value	0.483	− 0.934	− 1.173	− 0.339	− 0.527
H1	− 0.083	− 0.479	0.844	− 0.183	0.940
S1	0.486	0.571	− 0.588	0.405	− 0.369
B1	0.100	0.491	0.262	− 0.121	0.856
H2	0.437	− 1.107	− 0.662	− 0.642	0.119
S2	0.121	0.987	− 0.024	− 0.899	0.955
B2	0.057	0.562	− 1.090	0.708	− 1.315
H3	− 0.299	0.696	− 0.071	− 0.469	2.236
S3	− 1.901	1.426	− 0.158	− 0.276	− 1.194
B3	0.399	1.676	− 1.629	0.774	0.383

**Table 4:** Hidden layer to output layer connection weights and biases.

	*y*_1_ (poor)	*y*_2_ (excellent)
Offset value	0.710	− 0.895
*h*_1_	1.104	− 1.010
*h*_2_	1.416	− 1.484
*h*_3_	1.169	− 0.586
*h*_4_	− 0.254	0.626
*h*_5_	− 1.344	0.565

**Table 5:** Multi-layer perceptron neural network training correctness results.

Ideal ratings	Rating given by MLP		Correct rate
	Poor	Excellent	
Poor	166	28	85.6%
Excellent	36	64	64.0%

The prediction accuracy of the trained children's shopping cart product color decision model is shown in Table 5. The correct prediction rates in Table 5 are the correct rates given directly after the SPSS software MLP algorithm has finished training the decision model, which is the correct rate of Eq. (2) for the product colour decision. Equation (2) is trained from the MLP model, which has a tanh function as the hidden layer activation function and a softmax function as the output layer activation function. That is, the correct rates in Table 5 are supported by the tanh function and the softmax function. From Table 5, we can conclude that the trained children's shopping cart product color decision model has a very high recognition rate for the solutions rated as “poor” and also has a high recognition rate for the solutions rated as “excellent”, which can help designers in the subsequent color scheme screening work. It should be pointed out that the number of solutions in the validation group is relatively small due to the small number of animated images used in this paper. But the number of solutions in the validation group may be very large in the reality, so utilizing the trained intelligent algorithm for solution screening can effectively reduce the time costs.

1 $$ \left\{ \begin{aligned} h_{1} & = \tanh ( - 0.083x_{1} + 0.486x_{2} + 0.1x_{3} + 0.437x_{4} + 0.121x_{5} + 0.057x_{6} - 0.299x_{7} - 1.901x_{8} + 0.399x_{9} + 0.483) \\ h_{2} & = \tanh ( - 0.479x_{1} + 0.571x_{2} + 0.491x_{3} - 1.107x_{4} + 0.987x_{5} + 0.562x_{6} + 0.696x_{7} + 1.426x_{8} + 1.676x_{9} - 0.934) \\ h_{3} & = \tanh (0.844x_{1} - 0.588x_{2} + 0.262x_{3} - 0.662x_{4} - 0.024x_{5} - 1.090x_{6} - 0.071x_{7} - 0.158x_{8} - 1.629x_{9} - 1.173) \\ h_{4} & = \tanh ( - 0.183x_{1} + 0.405x_{2} - 0.121x_{3} - 0.642x_{4} - 0.899x_{5} + 0.708x_{6} - 0.469x_{7} - 0.276x_{8} + 0.774x_{9} - 0.339) \\ h_{5} & = \tanh (0.940x_{1} - 0.369x_{2} + 0.856x_{3} + 0.119x_{4} + 0.955x_{5} - 1.315x_{6} + 2.236x_{7} - 1.194x_{8} + 0.383x_{9} - 0.527) \\ y_{1} & = {\text{softmax}} (1.104h_{1} + 1.416h_{2} + 1.169h_{3} - 0.254h_{4} - 1.344h_{5} + 0.710) \\ y_{2} & = {\text{softmax}} ( - 1.010h_{1} - 1.484h_{2} - 0.586h_{3} + 0.626h_{4} + 0.565h_{5} - 0.895) \\ \end{aligned} \right. $$

In the formula, $x_{i}$ represents the variable input to the MLP model, which is the HSB value of the color; $h_{j}$ is the output value of the hidden layer of the model; And $y_{k}$ is the final output of the model.

### Validation of product colour decision model for children's shopping carts

In order to verify the effectiveness of the product color decision model for children's shopping carts, we input HSB data (Data in supplementary information) of the last 60 groups of color schemes (No.301–360) into the color decision model, and the scoring results are shown in Table 6. By comparing the score results in Table 6 with the subjective score results of the questionnaire (Data in supplementary information), it is found that the score results of the model and the subjective evaluation results have a high consistency (Of the 60 results predicted by the colour decision model, there were only 2 inconsistencies with the subjectively scored results), which indicates that the product color decision model of children's shopping cart is effective. Subjectivity plays an important role in color design and color decision making. Usually, other researchers also compare research results with subjective evaluation results to verify the validity of research results. If the subjective evaluation results are in good agreement with the research results, it represents the validity of the research^28,29^. Using this decision model, designers can obtain the optimal three-color combination that meets the needs of consumers, and can also design products in other fields according to the program of this study.

**Table 6:** Rating of MLP color schemes.

Program number	Forecast rating	Program number	Forecast rating	Program number	Forecast rating	Program number	Forecast rating	Program number	Forecast rating	Program number	Forecast rating
1	Excellent	11	Excellent	21	Poor	31	Excellent	41	Excellent	51	Poor
2	Poor	12	Poor	22	Excellent	32	Poor	42	Poor	52	Poor
3	Poor	13	Poor	23	Excellent	33	Poor	43	Excellent	53	Excellent
4	Poor	14	Poor	24	Poor	34	Excellent	44	Poor	54	Poor
5	Poor	15	Poor	25	Excellent	35	Poor	45	Poor	55	Excellent
6	Poor	16	Excellent	26	Poor	36	Poor	46	Poor	56	Poor
7	Poor	17	Poor	27	Poor	37	Poor	47	Poor	57	Poor
8	Excellent	18	Poor	28	Excellent	38	Excellent	48	Excellent	58	Poor
9	Poor	19	Excellent	29	Poor	39	Poor	49	Poor	59	Poor
10	Poor	20	Poor	30	Poor	40	Poor	50	Excellent	60	Poor

### Validation of the colour matching design method

In this paper, the product color design scheme obtained by using this color matching method is compared with the color scheme randomly selected by the designer to verify the effectiveness of the color matching design method based on eye-tracking technology and MLP. If the product color design scheme obtained by applying this design method has a higher score than that randomly selected by the designer, we will judge the proposed color matching method as effective. Usually, other researchers also compare the research results with the subjective evaluation results to verify the validity of the research results^28,29^. In this work, the designer randomly selects 6 product color design schemes rated as “excellent” based on the evaluation results of the product color decision model for children's shopping carts, and invite a novice designer to randomly generate 4 color schemes for the three-color children's shopping cart. Finally, 60 children are invited for the final validation.

**Figure 4:**
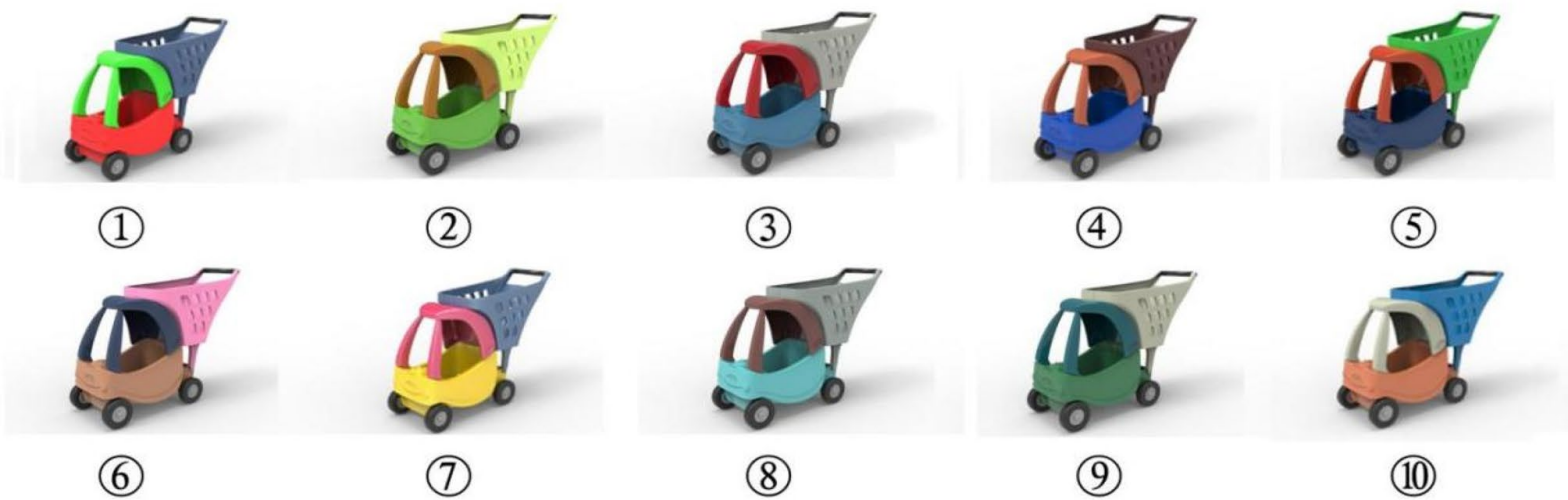
Validation of the three-color children's shopping cart color scheme used.

**Figure 5:**
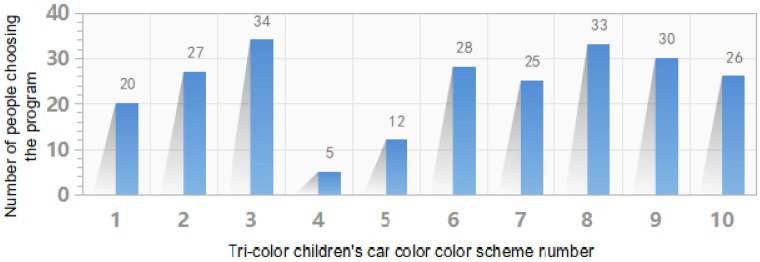
Summary of subjective findings in the experiment.

Firstly, we arrange the 6 color schemes obtained from the MLP color decision model and the 4 color schemes randomly generated by the novice designer in a chart in a random order, as shown in Fig. 4 (Fig. 4 was also generated by Keyshot 6.0 software). The images numbered 2, 3, 6, 8, 9, and 10 are the color schemes generated by extracting colors from the animated images, while the images numbered 1, 4, 5, and 7 are the color schemes randomly matched by the novice designer. Then Fig. 4 is displayed on a large screen in a kindergarten classroom and 60 children in 2 classes are asked to select the 4 product numbers they desire most from the 10 children's shopping cart products. The statistical results are collected in Fig. 5 (Fig. 5 is generated from the Excel application in Office 2019. Office 2019, https://www.microsoft.com/zh-cn/microsoft-365?rtc=1), where the color images numbered 2, 3, 6, 8, 9, 10 are desired by most people while color images numbered 1, 4, 5, and 7 are favored by little people. Experimental results demonstrate that the effectiveness and reliability of the color decision model based on the eye-tracking technology in converting animated image colors to product colors.

## Discussion and conclusion

This paper proposes a color matching design method from animations to products. Firstly, the colors from the animated images are extracted as the colors of the products based on eye-tracking technology. And then a color decision model for children's shopping cart products is established based on MIP, which provides designers with a wider range of color sources in the color design. The model assists designers to quickly and accurately screen and make decisions on product color schemes in various color combinations, thus greatly reducing the number of manual screening of schemes, improving the decision-making speed and providing a certain reference for color matching designers. Specific research results are as listed follows.

In this paper, an animature-to-product color design method is proposed, which combines eye-tracking technology with MLP algorithm. Among them, eye tracking realizes color extraction, MLP algorithm realizes color method screening, and the combination of the two completes the two most critical steps in color design. We found that the use of heat map in eye tracking technology instead of color clustering and other complex operations, the effect is very obvious. This method is more intuitive and accurate to extract the image color, for product color designers to provide more extensive color design sources and design reference. We also found that MLP algorithm can mine the implicit mapping relationship between color HSB value and user rating. Based on this mapping relationship, we constructed a product color decision model, which realized the classification and screening of a large number of product color combinations. Especially, when selecting schemes with poor rating, the accuracy was 85.6%. Eye tracking technology and MLP algorithm are an integral part of the whole product color design method. The combination of the two not only provides new ideas for the design of color design, but also avoids the designer to carry out multiple mathematical calculations. Importantly, it is friendly to the novice designer.

Equally importantly, the colour scheme approach based on eye tracking technology and MLP is not only applicable to non-real images, but also to real images. Some real images such as natural landscape images, animal images and product images may exhibit a higher level of complexity, which can cause problems for conventional colour extraction. In fact, eye-tracking techniques are less disturbed by factors such as complexity^30^. Although increasing the number of image colours reduces the efficiency of visual search, it does not affect the results of colour extraction by eye-tracking techniques^31^. In addition, multilayer perceptron neural networks (MLP) have the advantages of being adaptive, self-learning, real-time and highly robust, and are equally applicable to the classification and selection of other image colour schemes^21^.

It should also be noted that we propose a colour scheme design method from animation to product that combines eye tracking with MLP algorithms, which has some unique advantages compared to similar methods. For example, Shiguang Liu et al. used physical fabric images for fabric colour design^32^, and Ali Jahanian et al. used a palette to extract pixels from prominent regions in animal images^33^. The difference is that we extend the sources of product colour design by using a non-real image colour. Importantly, our colour extraction uses eye-tracking technology, which extracts a more intuitive map of heat zones, thus avoiding the need for formulas such as colour clustering and reducing the requirement for expertise in product colour design^11,16,19,21^. In addition, they also established the sentiment relationship between target and candidate themes through K-means algorithm and emotional entropy algorithm, and proposed a new colour design approach^32,34^. In our study, a supervised MLP algorithm is used to train the color decision model, and the implicit mapping relationship between color and user rating is mined, so as to realize the selection and decision of a large number of color combinations. This is also in line with the suggestion of Gianluigi Ciocca et al. in the study of color extraction, who compared several state-of-the-art methods for extracting color from images and found that supervised algorithms have advantages over unsupervised ones^35^.

Nevertheless, we should highlight two limitations in our study. On the one hand, although it is possible to extract colours of interest to subjects in images using eye-tracking techniques, eye-tracking devices do entail a certain monetary cost, which is not friendly to the average designer. On the other hand, we used the well-established Multilayer Perceptron Neural Network (MLP) algorithm in SPSS software to realize the selection and decision of a large number of product color combinations, but the results of the decision did not exactly give an optimal solution. In short, the colour decision model trained in this study can only give excellent, medium and poor ratings, and when there are many excellent solutions with little difference, the designer may still experience some choice anxiety. Therefore, some more powerful mathematical algorithms are also worth considering. For example, Artificial Neural Network (ANN) and Convolutional Neural Networks (CNN) can enable deeper mining of mapping relationships.Despite these limitations, this study further expands the sources of image colour in product colour design, and the potential contribution of using eye-tracking techniques for the extraction and reuse of animated image colour in product colour matching remains important, providing a more solid basis for future colour research^36^.

In the future, we will aim to apply eye-tracking techniques to all stages of product colour design for in-depth discussion and consider more powerful mathematical algorithms for colour design to improve the science of product colour design.

## Materials and methods

### Experimental materials

**Figure 6:**
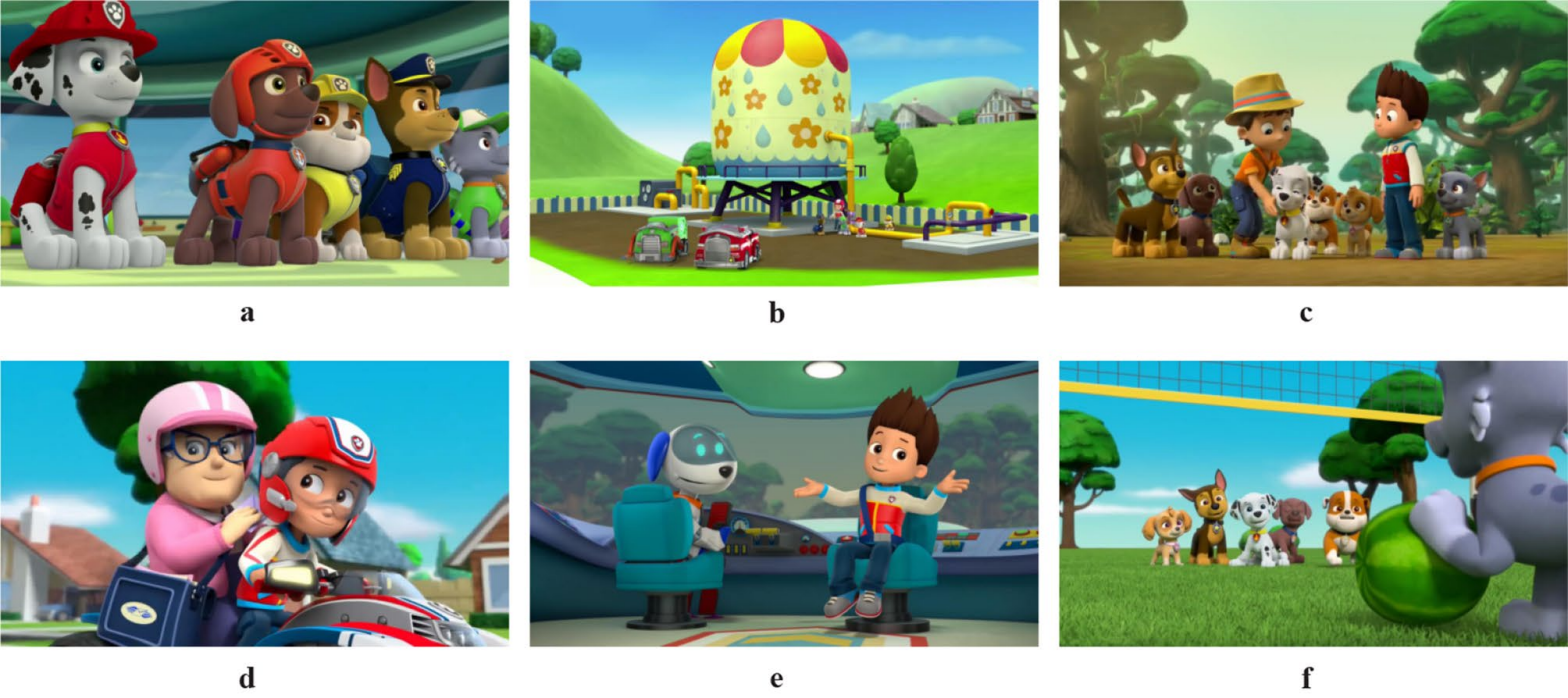
PAW Patrol animation images. © publicly available animated image from the Mango TV platform.

In this study, six animated images from the children's animated images of “PAW Patrol” are selected as the material for color extraction. Those images were randomly selected in the form of animated screenshots from the main episodes of episodes 1, 17, 33, 58, 76, and 99, which are shown in Fig. 6. Figure 6 is a publicly available animated image from the Mango TV platform. (Mango TV, https://www.mgtv.com/b/312567/3846522.html?fpt=1&ftl=2). As Children's favorite entertainment, animation play an important role in the process of children's growth^12^. “PAW Patrol” is an action-adventure cartoon series launched by Nickelodeon, which tells the story of a 10-year-old boy, Ryder, who sets up a rescue team with his adopted puppies to help people. The captain in “PAW Patrol” takes on the leader responsibility and has an almost perfectly positive persona, acting as a moral benchmark as well as a code of conduct. The members in “PAW Patrol” become favorite images that children like in hearts with the teamwork spirit of conveying love, responsibility and mutual help. Particularly, the colors of the animated images of “PAW Patrol” leave a deep impression on children^37^.

**Figure 7:**
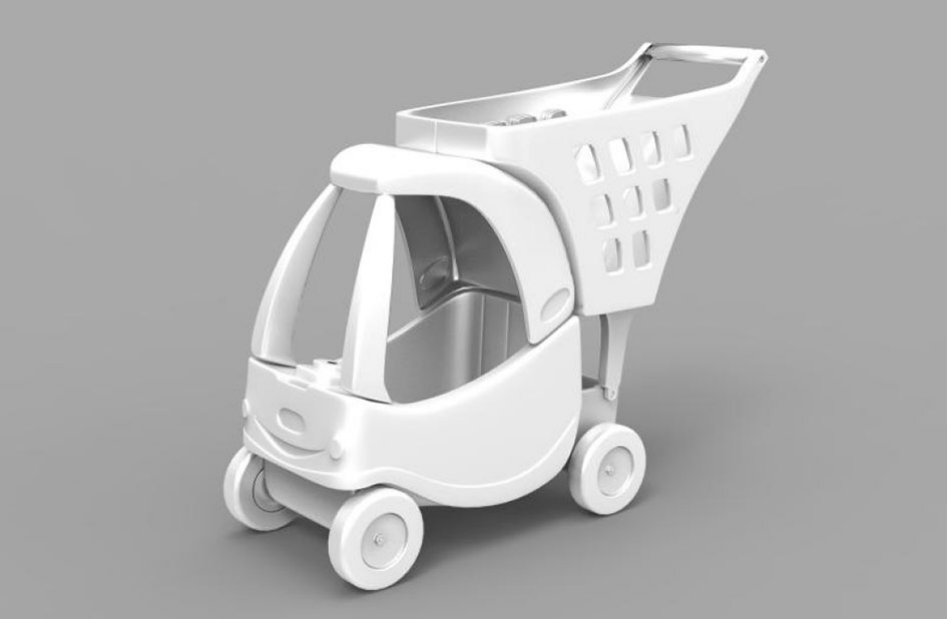
The 3D model of children's shopping cart.

In this paper, the children's shopping cart model with three main colors is selected as the color design product, whose 3D model is shown in Fig. 7 (Fig. 7 was also generated by Keyshot 6.0 software). The reasons are as follows. On one hand, more than 20,000 children are injured by adult shopping carts and sent to the emergency room each year in the United States, with most of the injuries occurring in the face and neck. Therefore, professionally designed shopping cart products for children are valued by designers (Supermarket shopping cart)^38^. On the other hand, the users who watch the animation of “PAW Patrol” match the age of users of children's shopping cart products. From the emotional needs of children while watching animation, mapping the “PAW Patrol” animation colors to the color scheme of children's shopping cart products helps to improve children's product experience and shopping experience^39^. In practical product design, designers usually choose three, five or seven colours for product design, and three colours are widely adopted and given a certain colour share because the colours of the product then appear clean and balanced, rather than busy and cluttered^28^. In scientific research, tricolour is more likely to elicit feelings from subjects than monochrome and bicolour, and is also more conducive to reducing the work of theoretical research than five and seven colours, making tricolour products the most valuable for practical research and widely adopted in colour research^29^. It is important to note that even when the colour type of the product is increased to five or seven colours, our method still works because of its flexibility. Even with the increase of color types, MLP can still assist designers to complete the task of color matching, which reflects the real-time, intelligent and adaptive of MLP^21,33^. In addition, the three-color children's shopping cart can not only attract children's attention, but also arouse children's happy feeling when three colors are carefully combined^2,13^.

### Methodology

The study was validated and approved by The Biomedical Ethics Committee of Anhui University (BECAHU-2022-003), and all studies were conducted in accordance with the Declaration of Helsinki and other relevant guidelines, and all experimental subjects voluntarily signed the informed consent form after being fully informed before conducting the experiments and subjective studies. All subjects and/or their legal guardians have informed and consented to the publication of identifying information/images in online open access publications. All experimental subjects voluntarily signed the informed consent from their legal guardian or parent form after being fully informed before conducting the experiments and subjective studies.

#### Eye-tracking technology

The eye-tracking technology is based on infrared devices and image acquisition devices, which tracks subtle changes in eye features in real time by actively projecting light beams such as infrared rays to the iris. The eye-tracking device used in this study was the Tobii Pro Glasses 2, manufactured in Sweden. The eye-tracking technique has less interferes with the acquisition of the subject's evaluation process and meanwhile enables the recording of various data during eye movements^30^.

There are four common methods for data visualization of eye-tracking: AOI method, scan path method, 3D spatial method and thermal zone map method. The hot zone map method is selected for color extraction of animated images. The eye-tracking data visualization method should be reasonably determined according to the actual design evaluation content^30,31,40–42^. In the heat zone map method, the denser the hot spots, the longer the subject's gaze time in the area of interest, the longer the gaze time, and the greater the interest in the color matching of the area^43^. Therefore, extracting colors and obtaining color schemes by the heat zone map method of eye-tracking experiments is in line with the expected goal of this study^14^.

#### Multilayer perceptron neural network

Multilayer perceptron neural network (MLP) can be regarded as a logistic regression classifier, which consists of three parts: the input layer, the hidden layer, and the output layer. The input layer receives the external data and calculates the excitation values through the activation function, and then the values are passed to the hidden layer. The hidden layer takes the results from the upper layer as input and calculates the excitation values through the activation function, and the obtained data are passed to the output layer. The nodes of the hidden layer and the output layer can be perceptrons. The multilayer perceptron with more nodes has multiple perceptrons and the output layer can also be adjusted according to the actual application^44^.

The output layer in MLP often uses the soft max function in many color combination screening or classification tasks. The mathematical expression of MLP is shown in Eq. (2):

2 $$ \left\{ \begin{gathered} h_{j} = \varphi^{(1)} \left( {\sum\limits_{i = 1}^{n} {x_{i} \cdot \omega_{ji}^{(1)} } + b^{(1)} } \right) \hfill \\ y_{k} = \varphi^{(2)} \left( {\sum\limits_{j = 1}^{n} {h_{j} \cdot \omega_{kj}^{(2)} } + b^{(2)} } \right) \hfill \\ \end{gathered} \right. $$

where *m*, *n* and *k* represent the numbers of neurons in the input layer, the hidden layer and output layer, respectively. $h_{j}$ is the output value of the hidden layer, $\varphi^{(1)}$ refers to the activation function from the input layer to the hidden layer, $x_{i}$ denotes the external input data, $\omega_{ji}^{(1)}$ is the connection weight from neurons in the input layer to neurons in the hidden layer, $b^{(1)}$ is the bias value of the hidden layer,$y_{k}$ is the output value of the output layer, $\varphi^{(2)}$ is the activation function from the hidden layer to the output layer, $\omega_{kj}^{(2)}$ is the connection weight from neurons in the hidden layer to neurons in the output layer, $b^{(2)}$ is the activation function from the hidden layer to the output layer^37^.

This work adopts an MLP to deal with the mapping relationship problem between user ratings and color combinations for the preparation of multiple color combination selection. The relationship between dependent variables and independent variables in the product color decision is not linear. The MLP is the core of machine learning and deep learning, which has the advantages of self-adaptability, self-learning, real-time, high robustness and so on. Multiple hidden layers facilitate scholars to build powerful models for efficiently solving nonlinear system problems^21^. All the above studies used MIPs for screening and decision making of practical problems, and the experimental findings demonstrated that the MIP has substantial improvement in stability and accuracy compared with other algorithms^45–47^. Therefore, screening multiple color combinations for children's shopping carts by MIPs is in line with the expected goal of this study.

## Data Availability

All the datasets generated or analyzed during the study are included in this published article and its supplementary information.
